# TLR4/CD14/MD2 Revealed as the Limited Toll-like Receptor Complex for *Chlamydia trachomatis*-Induced NF-κB Signaling

**DOI:** 10.3390/microorganisms10122489

**Published:** 2022-12-15

**Authors:** Romana Klasinc, Claire Battin, Wolfgang Paster, Michael Reiter, Philipp Schatzlmaier, Peter Rhein, Andreas Spittler, Peter Steinberger, Hannes Stockinger

**Affiliations:** 1 Medical University of Vienna, Center for Pathophysiology, Infectiology and Immunology, Institute for Hygiene and Applied Immunology, 1090 Vienna, Austria; 2 Medical University of Vienna, Center for Pathophysiology, Infectiology and Immunology, Institute of Immunology, Division of Immune Receptors and T Cell Activation, 1090 Vienna, Austria; 3 St. Anna Children’s Cancer Research Institute (CCRI), 1090 Vienna, Austria; 4 Luminex B.V., A DiaSorin Company, 5215 MV ‘s-Hertogenbosch, The Netherlands; 5 Medical University of Vienna, Core Facility Flow Cytometry and Department of Surgery, Research Laboratories, 1090 Vienna, Austria

**Keywords:** toll-like receptors, *Chlamydia trachomatis*, signaling, imaging, flow cytometry

## Abstract

*Chlamydia trachomatis (Ct)* is the most common cause of genital tract infections as well as preventable blindness worldwide. Pattern recognition receptors such as toll-like receptors (TLRs) represent the initial step in recognizing pathogenic microorganisms and are crucial for the initiation of an appropriate immune response. However, our understanding of TLR-signaling in *Chlamydia*-infected immune cells is incomplete. For a better comprehension of pathological inflammatory responses, robust models for interrogating TLR-signaling upon chlamydial infections are needed. To analyze the TLR response, we developed and utilized a highly sensitive and selective fluorescent transcriptional cellular reporter system to measure the activity of the transcription factor NF-κB. Upon incubation of the reporter cells with different preparations of *Ct*, we were able to pinpoint which components of TLRs are involved in the recognition of *Ct*. We identified CD14 associated with unique characteristics of different serovars as the crucial factor of the TLR4/CD14/MD2 complex for *Ct*-mediated activation of the NF-κB pathway. Furthermore, we found the TLR4/CD14/MD2 complex to be decisive for the uptake of *Ct*-derived lipopolysaccharides but not for infection and replication of *Ct*. Imaging flow cytometry provided information about inclusion formation in myeloid- as well as lymphocytic cells and was highest for *Ct* L2 with at least 25% of inclusion forming cells. *Ct* E inclusion formation was eminent in Jurkat cells without CD14 expression (11.1%). Thus, our model enables to determine *Ct* uptake and signal induction by pinpointing individual components of the recognition and signaling pathways to better understand the immune response towards infectious pathogens.

## 1. Introduction

*Chlamydia trachomatis* (*Ct*) is an intracellular pathogen presenting a diverse spectrum of human diseases. It is recognized as the most prevalent sexually transmitted bacterial infection globally with estimated 131 million new cases annually among adults [1,2]. *Ct* is known as the etiologic agent of urogenital and ocular infections that are linked to distinct serovars of this pathogen. The serovars A, B, Ba and C are agents of trachoma (also known as preventable blindness); serovars D, Da, E, F, G, H, I, Ia, J and K are agents of non-invasive urogenital, oropharyngeal, anorectal and ocular infections; and serovars L1, L2, L2a and L3 are agents of lymphogranuloma venereum (LGV) [1,3,4]. LGV presents with invasive urogenital and anorectal infections, but the mechanisms why LGV strains manifest distinct symptoms compared to the oculo-genital strains are poorly understood. A possible explanation is the dissemination through the lymphatic system by invasion of dendritic cells (DCs) or macrophages, which drain into the lymphatic system [5,6,7]. The epidemiology of LGV differs between endemic (in South America, Southeast Asia, Middle East, and Africa) and non-endemic cases among men, who have sex with men, with a prevalence of approximately 1% in the latter [8]. In contrast, non-invasive urogenital strains appear mainly asymptomatic as only 13% of women tested positive for *Ct* presented with cervical discharge [9]. Complications associated with an infection can manifest as pelvic inflammatory disease with potentially life-threatening tubo-ovarian abscesses, or extraarticular manifestations such as urethritis and salpingitis resulting in infertility [4].

The innate immune system represents the first line-defense in chlamydial infections and therefore crucially shapes the following immune responses. Here, the recognition of *Ct* by pattern-recognition-receptors (PRRs) contributes to a significant proportion in initiating the immune response towards this organism. Previously described important PRRs in chlamydial infections are toll-like-receptors (TLRs), NOD-like-receptors (NLRs) and cytoplasmatic DNA/RNA sensors. The stimulation of these receptors and sensors induces recruitment of immune cells to the site of infection and consequently plays an important role in shaping the infection [10,11]. Several studies reported that recognition and signaling of *Ct* was preferentially mediated by TLR2 [12,13,14]. However, the recognition of lipopolysaccharide (LPS) from Gram-negative bacteria is mediated by TLR4 in complex with the myeloid differentiation factor 2 (MD2) and the transfer of LPS to this complex is provided by accessory molecules, i.e., either LPS-binding protein or CD14 [15]. Similar to other Gram-negative bacteria, *Ct* membranes also contain LPS. Though, the role of the TLR4/MD2 complex in recognizing *Chlamydia* LPS was discussed controversially. First, the structure of chlamydial LPS differs from typical enterobacterial LPS and with this structural difference its immunogenic potential is also modified. Some studies suggested chlamydial LPS to have a 100 times less potent activity than LPS from *Escherichia coli* [16,17,18]. Whether this attenuated immune activation potential is part of an escape mechanism or an immunomodulation has to be determined and, therefore, the importance of immune recognition of chlamydial LPS must not be neglected. In this respect, several studies lined out the importance of TLR4 for *Ct* recognition by the host cell or clearance of *Ct* infection [19,20,21,22]. However, whether *Ct*-LPS is involved in this process is unresolved, as well as whether serovar-specific differences in TLR4 recognition are associated with variations in the immune response and consequently with distinct clinical manifestations [13,16,23].

In this study, by implementing a TLR/NF-κB cellular reporter system, we show that CD14 is the decisive factor of the TLR4/CD14/MD2 complex for induction of the NF-κB pathway by *Ct*. Furthermore, we demonstrate the complete TLR4/CD14/MD2 complex to be essential for *Ct*-LPS uptake by cells, but not for uptake and infection of intact *Ct*.

## 2. Materials and Methods

### 2.1. Microbial Strains and Ct Culture

The *Ct* serovars E (DSM 19131) and L2 (DSM 19102) used in this study were propagated either in HeLa human epithelial cells (ATCC^®^ CCL-2^TM^) or McCoy [McCoyB] mouse fibroblasts (ATCC^®^ CRL-1696^TM^) with modifications, as previously described [24,25].

### 2.2. Cell Lines and Culture Conditions

The human monocytic cell line THP-1 [26] and the human lymphoblastic T-cell line Jurkat, clone E6-1, were maintained in Gibco™ RPMI 1640 medium, GlutaMAX™ Supplement (Thermo Fisher Scientific, Waltham, MA, USA), supplemented with 10% heat-inactivated fetal calf serum (FCS; Biowest, Nuaillé, France). In brief, the THP-1- and Jurkat T-cells were retrovirally transduced with the NF-κB-eGFP construct and eGFP-low expression cells were generated as described [27,28]. To obtain stable expressing NF-κB-eGFP reporter cell lines, from these eGFP-low expressing cells, single cell clones were established. The generation of the TLR-sensitive NF-κB-eGFP reporter cells encoding human TLR2/1, TLR2/6, TLR4, CD14 and MD2 was also recently described [28,29]. To differentiate between CD14 dependent and independent TLR4 signaling, the Jurkat NF-κB-eGFP reporter cells were equipped with TLR4/MD2 with or without the CD14 co-receptor. The cell lines with different sets of TLRs used in our experiments are shown in Figure S1. All cells were cultured in a humidified atmosphere of 5% CO_2_ at 37 °C and routinely tested for mycoplasma contamination.

### 2.3. Reagents and Antibodies

Agonists for TLR1/2 (Pam3CSK4, synthetic triacylated lipopeptide), TLR2/6 (FSL-1, synthetic diacylated lipopeptide), TLR4 (*E. coli* LPS-EB ultrapure) and TLR5 (Flagellin) were purchased from Invivogen (San Diego, CA, USA). Phorbol-12-myristat-13-acetat (PMA) and ionomycin were obtained from Sigma Aldrich (St. Louis, MO, USA). Lyophilized *Ct*-LPS Typ L2 and Typ E were both purchased from Glycobiotech (Kuekels, Germany). For intracellular staining of *Ct* within reporter cell lines the anti-*Chlamydia* lipopolysaccharide (*c*LPS) monoclonal antibody (mAb) 512F (Life Technologies, Carlsbad, CA, USA) was conjugated with Alexa Fluor 647 (AF647) and purified by fast protein liquid chromatography. Live/dead staining was performed using Zombie Aqua™ or Zombie Yellow^TM^ fixable viability dye (both BioLegend, San Diego, CA, USA). AF647-conjugated anti-human TLR5 mAb, clone 624915 (Bio-Techne, Minneapolis, MN, USA), APC-conjugated CD283 (TLR3) mAb, clone TLR 3.7, APC-conjugated CD286 (TLR6) mAb, clone REA382, VioBlue-conjugated CD14 mAb, clone REA599 (all Miltenyi Biotech, Bergisch Gladbach, Germany), APC-conjugated CD14 mAb, clone M5E2 (BioLegend), APC-conjugated CD284 (TLR4) mAb, clone HTA125, unlabeled TLR4 mAb, clone W7C11 (InvivoGen, Toulouse, France) with APC-conjugated goat F(ab’)2 anti-mouse IgG (Jackson. ImmunoResearch, West Grove, PA, USA), AF647-conjugated CD282 (TLR2) mAb, clone TLR2.1 (both BioLegend) and AF647-conjugated TLR1 (H-8): sc-514399 (Santa Cruz Biotechnology, Santa Cruz, CA, USA) were used to verify the TLR expression profiles of the reporter cell lines. 

### 2.4. Stimulation of Reporter Cells

The reporter cells were cultured in the presence of stimuli for 20 or 40 h. Assays were performed in either 96-well or 24-well flat bottom plates - depending on the number of cells needed for the analysis - at a concentration of 1 × 10^6^ cells per mLin a total volume of 100 μL or 1000 µL (including stimulus), respectively. Stimuli added to the cell culture were *Ct*-LPS from serovar L2 (*Ct*-LPS-L2) and E (*Ct*-LPS-E), viable *Ct* at a multiplicity of infection (MOI) of 10 or 1, as well as UV-inactivated [MOI 10 (UV)], or azithromycin (AZT) treated *Ct* [MOI 10 (AZT)] preparations. For mock controls, the cells were treated with medium only without stimuli. In addition, depending on the experiment, different TLR agonists were added as controls. Cells were then harvested and eGFP expression as well as the signal intensity after intracellular staining with cLPS-specific AF647-conjugated mAb 512F were analyzed by either flow cytometry or imaging flow cytometry. Mean and standard deviation of mean fluorescence intensity (MFI) of the viable population of reporter cells were determined. All samples were analyzed in duplicates, unless indicated otherwise.

### 2.5. Analysis by Flow Cytometry

The cells were washed with staining buffer [PBS supplemented with 1% BSA (Carl Roth, Karlsruhe, Germany) and 0.02% NaN3], and nonspecific binding of the mAbs to Fc-receptors was prevented by blocking with 2.4 mg/mL human IgG (Beriglobin P; CSL Behring, King of Prussia, PA, USA) on ice for 30 min. Then, mAb/fluorochrome conjugates or appropriate isotype controls were added. Cells were incubated for 30 min on ice and washed twice with staining buffer. For the intracellular staining protocol, cells were first stained with the fixable viability dyes, then fixation was performed with 4% paraformaldehyde (PFA) for 15 min, followed by permeabilization with 0.1% saponin buffer. The intracellular staining was performed for 30 min with the AF647-conjugated cLPS mAb for detection of intracellular *Ct*. The samples were analyzed according to the guidelines for the use of flow cytometry [30] on either a LSR II flow cytometer (BD Biosciences, Franklin Lakes, NJ, USA) or on the Cytek Aurora (Cytek Biosciences, Inc., Fremont, CA, USA). Living cells were gated according to their forward and side scatter characteristics and by excluding dead cells using fixable viability dyes. The data were processed with the FlowJo software (Tree Star, Ashland, OR, USA). For the statistical analysis and generation of graphs Prism V7 (GraphPad Software, San Diego, CA, USA) was used. Statistical significance was determined between the groups with a two-way analysis of variance (2way ANOVA) followed by Dunnett’s or Tukey’s multiple comparisons test. Significance was set at a *P* value of less than 0.05.

### 2.6. Infectivity Assay of Reporter Cells using Imaging Flow Cytometry 

*Ct* replication and survival were measured by imaging flow cytometry (Amnis^®^ ImageStream^®X^ MkII, Luminex, Austin, TX, USA) to visualize *Ct* inclusions. The infectivity assay, the sample preparation, and the staining with mAbs were carried out in the same way as for flow cytometry but at higher cell concentrations (500,000 to 1,000,000 cells diluted in 50 µL staining buffer). The versatile utilization of the imaging flow cytometer for analysis of intracellular pathogens was previously described [31,32]. Samples were acquired at 60× magnification using the integrated software INSPIRE™ (version 200.1.620.0 Luminex) for data collection. The internalization assay was quantified by using image-based algorithms in the ImageStream^®^ Data Exploration and Analysis Software (IDEAS^®^ 6.2.187, Luminex). The analysis was restricted to single cells in best focus. Single cells were identified by their intermediate size (area) and high aspect ratio (minor axis divided by the major axis) in comparison to debris (small area and a range of aspect ratios depending on the shape of the debris) and coincident events (large area and small aspect ratio). Out-of-focus cells were excluded by using the feature Brightfield Gradient RMS, a measurement of image contrast. Only cLPS positive cells with a max pixel intensity of over 100 arbitrary units (a.u.) were selected to quantify the internalization of *Ct*. To calculate the percentage of cells with internalized *Ct*, the following mask was designed that identified the inside of the cells. First, the Tight Object Mask of the brightfield image was used to define the area corresponding to the cell. Afterwards, this mask was eroded by 4 pixels to exclude the cell membrane. The intracellular mask was then used to calculate the internalization feature applied to the cLPS AF647 channel. Internalization was defined as the ratio of the cLPS intensity inside the cell (the intracellular mask) to the intensity of the entire cell. This ratio was log transformed to increase the dynamic range (−infinity to +infinity). Cells that had internalized *Ct* had positive scores while cells with little internalization had negative scores. Two morphologically different populations, one consistent with large *Ct* inclusions, the other with undeveloped or “small inclusions – spots” were identified in the data sets. The algorithm-based discrimination between spots and inclusions was achieved by implementing the Feature Finder wizard of the IDEAS^®^ software. After manual selection of the two “truth” populations, the Feature Finder algorithm calculated the Fisher’s Discrimination Ratio (Rd) metric for a selection of single features to quantify the separation power between the two populations, by considering the mean (µ) and the standard deviation (σ).

$$Rd=\frac{inter-class\;variance\;}{intra-class\;variance}=\frac{µ\;\left(positive\;truth\right)-µ\;\left(negative\;truth\right)}{\sigma \;\left(\;positive\;truth\right)+\sigma \;\left(negative\;truth\right)}$$

## 3. Results

### 3.1. Identification and Analysis of TLRs for Ct Recognition

**Figure 1:**
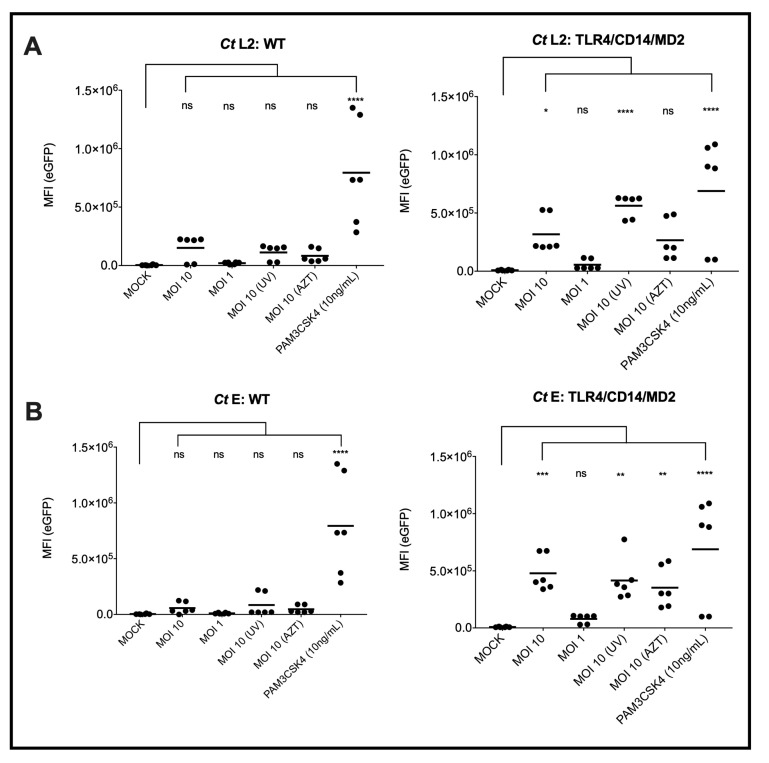
NF-κB induced expression of eGFP by *Ct* in WT or TLR4/CD14/MD2 THP-1 reporter cells. The eGFP-MFI of three independent experiments was measured 20 h post inoculation of the cells with *Ct serovar* L2 (**A**) or E (**B**). PAM3CSK4 was used as control for a TLR4/CD14/MD2 independent activation of the reporter cells by naturally expressed TLR1/2. Statistical differences between mock and different conditions were assessed by 2way ANOVA followed by Dunnett’s multiple comparisons test. * *p* < 0.05, ** *p* < 0.01, *** *p* < 0.001, **** *p* < 0.0001; ns, not significant. Individual measurements are shown (n = 6), each experiment was performed in duplicates.

To identify and analyze the signaling function of the different cell surface TLRs that recognize bacterial cell wall products, i.e., TLR1/2, TLR2/6, TLR4, TLR4/CD14 and TLR5 upon recognition of *Ct*, we used the monocytic cell line THP-1 and the T-cell line Jurkat. Both cell lines, previously described as potential host cells for *Ct* serovar L2 [33,34], were transduced with a construct encoding eGFP under the control of a NF-κB response element as described earlier by us [27]. THP-1 cells express constitutively TLR1/2, TLR2/6 and TLR5, accordingly, the reporters responded towards the corresponding TLR ligands Pam3CSK4, FSL-1 and flagellin in the picomolar range [28,29]. To complete their TLR surface profile, we transduced THP-1 cells with the TLR4/CD14/MD2 complex that made them sensitive to *E. coli* LPS in the pg/mL range [28]. Upon incubation with *Ct* serovars L2 and E either in a viable or UV-treated form at MOI 10, eGFP was only significantly induced in the THP-1 reporter cells expressing the TLR4/CD14/MD2-complex (Figure 1). The TLR1/2 agonist Pam3CSK4 induced eGFP also in the wild-type (WT) THP-1 cells confirming that the NF-κB eGFP construct is functional in these cells as previously shown by us [27]. When compared to the mock-treated cells, the AZT preparation of serovar E induced a significant response in contrast to L2 (Figure 1A,B). 

**Figure 2:**
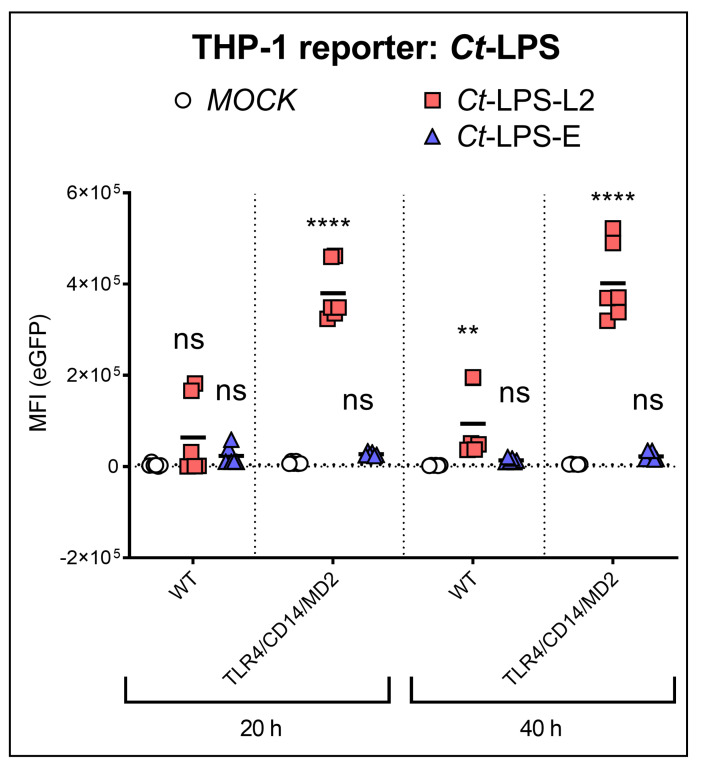
NF-κB induced expression of eGFP upon recognition of Ct-LPS (500 ng/mL) derived from serovar L2 or -E. The experiments were performed after 20 h or 40 h of incubation with WT- or TLR4/CD14/MD2 THP-1 reporter cells. The data represent eGFP-MFI means ± SEM of three independent experiments. Statistical differences between WT (20 h) and individual conditions were assessed by 2way ANOVA followed by Dunnett’s multiple comparisons test for each serovar. ** *p* < 0.01, **** *p* < 0.0001; ns, not significant. Individual measurements are shown (n = 6), each experiment was performed in duplicates.

A further difference between serovars L2 and E, which we found with the pure monomeric *Ct*-LPS preparations, was that the TLR4/CD14/MD2 THP-1 reporter cells were only activated by *Ct*-LPS derived from serovar L2. Even after an incubation time of 40 h, *Ct*-LPS-E did not induce eGFP expression (Figure 2).

### 3.2. Identification and Analysis of TLRs for Ct Recognition

**Figure 3:**
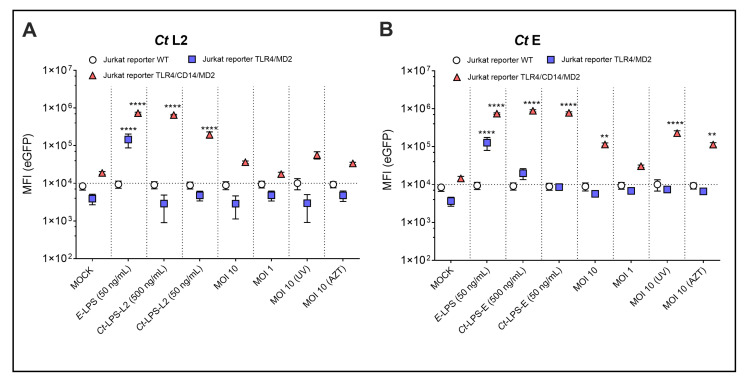
NF-κB dependent expression of eGFP upon incubation of different Jurkat reporter cells with various LPS- and *Ct*-preparations. WT, TLR4/MD2 or TLR4/CD14/MD2 Jurkat reporter cells after 20 h of incubation with *Ct* serovar L2 (**A**) or serovar E (**B**), E-LPS–*E. coli* derived LPS, *Ct*-LPS-L2–*Ct* serovar L2 derived LPS, *Ct*-LPS-E–*Ct* serovar E derived LPS, MOI 10 (UV)–UV-treated *Ct* with MOI 10, MOI 10 (AZT)–AZT-treated *Ct* with MOI 10. The data represent eGFP-MFI means ± SEM of three independent experiments. Statistical differences were assessed by 2way ANOVA followed by Dunnett’s multiple comparisons test. ** *p* < 0.01, **** *p* < 0.0001. Only statistically significant differences between conditions are indicated. Error bars represent mean ± SEM.

To confirm the requirement of the TLR4/CD14/MD2 complex for NF-κB signal induction by *Ct*, we used Jurkat T-reporter cells. Jurkat T-cells constitutively express functional TLR5 only [29]. We ectopically expressed either TLR4/MD2 alone or together with CD14 (Figure S1). Both *Ct* serovars L2 and E induced eGFP expression only in the TLR4/CD14/MD2 expressing Jurkat reporters, demonstrating the strong dependency on the CD14 co-receptor for *Ct* recognition (Figure 3). The eGFP signal intensity was weak for the viable as well as the UV- and AZT-treated preparations, but strong after incubation with monomeric *Ct*-LPS. In contrast to the THP-1 reporter cells, the Jurkat reporter cells responded not only towards *Ct*-LPS-L2 (Figure 3A) but also towards *Ct*-LPS-E (Figure 3B), and the eGFP of the latter signal was approximately four times higher than that of *Ct*-LPS-L2 (eGFP-MFI: 7.81 × 10^5^ versus 1.95 × 10^5^) and comparable to the signal of *E. coli* LPS at the same concentration (eGFP-MFI: 7.46 × 10^5^). In contrast to *Ct*-LPS of both serovars, *E. coli* LPS was able to activate the Jurkat reporter cells expressing TLR4/MD2 only, confirming the different recognition of *E. coli* LPS and *Ct-*LPS on the one hand [16] and the functionality of the TLR4/MD2 transductant on the other hand. When analyzing NF-κB signaling in the TLR4/CD14/MD2 Jurkat reporter cells after an incubation of 40 h, we found a significant increase with viable *Ct* serovar L2 (MOI 10 and MOI 1) (Figure S2A), but a less pronounced activation with *Ct* serovar E (Figure S2B).

The THP-1 reporter cells already suggested that neither TLR1/2 nor TLR 2/6 are involved in NF-κB activation by *Ct*. We confirmed this assumption by ectopically expressing either TLR1/2 or TLR 2/6 in the Jurkat reporter cells. Although these reporters responded in the picomolar range towards their respective TLR ligands Pam3CSK4 and FSL-1 [28,29], neither *Ct* serovar L2 (data not shown) nor serovar E (Figure S3) induced a significant eGFP signal in these reporters.

### 3.3. Simultaneous Analysis of Infectivity and Signal Induction by Ct

**Figure 4:**
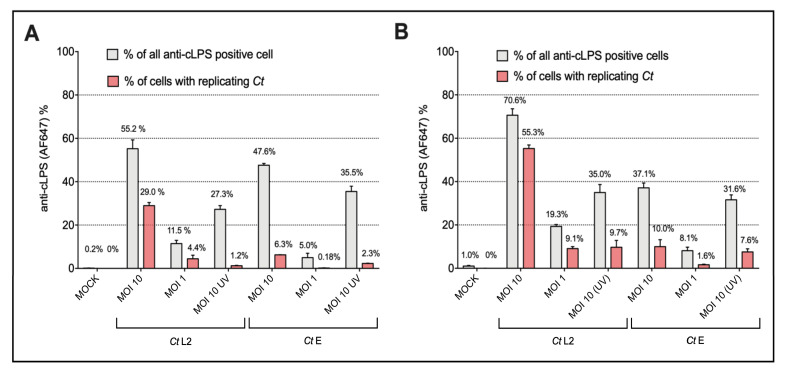
Flow cytometric analysis of anti-cLPS positive (**A**) THP-1 TLR4/CD14/MD2 and (**B**) Jurkat TLR4/CD14/MD2 reporter cells. The cells were incubated for 40 h with *Ct* preparations of serovar L2 (*Ct* L2) or serovar E (*Ct* E). The preparations included viable bacteria (MOI 10, MOI 1) as well as UV- [(MOI 10 (UV)] *Ct*. Mock controls are shown on the left. The grey bars indicate percentages of cLPS positive cells in the upper two contour plot quadrants of the flow cytometry plots. The red bars represent percentages of cells localized in the red gate that contain with high probability replicating *Ct*. The flow cytometry plots of one representative experiment are shown in Figure S4. The data represent percentage means (numbers shown) ± SEM of three independent experiments.

Because *Ct* preparations induced NF-κB signaling only in the THP-1- and Jurkat reporters expressing TLR4/MD2 plus CD14, we used these cells to simultaneously analyze by flow cytometry not only signaling by NF-κB-induced expression of eGFP but also uptake of *Ct* by intracellular staining using an anti-cLPS antibody. Besides viable *Ct*, we also used UV-inactivated and AZT-treated *Ct* samples. In addition, we analyzed the uptake and signaling of monomeric *Ct*-LPS derived from both serovars (Figure 4 and Figure S4). In terms of uptake, a serovar specific difference was evident, which was similar for both cell lines. Staining with the cLPS mAb showed that viable *Ct* serovar L2 at MOI 10 and MOI 1 forms a clearly defined separate population depicted in the red gate in Figure S4 and displayed by the red bars in Figure 4. This distinct population was not seen when using the UV- or AZT-treated L2 preparations. In contrast, no differences were detectable between viable, UV- or AZT-treated *Ct* preparations of serovar E, erroneously suggesting that *Ct* serovar E replicates neither within the lymphocytic nor the monocytic reporter cells. When measuring signal induction by NF-κB-induced expression of eGFP in the same cells, we found not only a serovar, but also a cell line dependency. In contrast to the THP-1 TLR4/CD14/MD2 reporter cells (Figure 1), in the Jurkat TLR4/CD14/MD2 reporter cells, only serovar E and not L2 induced NF-κB signaling after 20 h of incubation (Figure 3); however, L2 induced a significant activation upon a 40-h incubation (Figure S2). Further, Jurkat reporter cells equipped with the TLR4/CD14/MD2 complex reacted significantly differently towards monomeric *Ct*-LPS (L2 was four times less active than E), compared to the THP-1 TLR4/CD14/MD2 reporter cells that got activated only by *Ct*-LPS-L2 (Figure 2 and Figure 3).

### 3.4. Identification and Enumeration of Ct-Infected Cells by Imaging Flow Cytometry

As shown above by standard flow cytometry, inoculation of the reporter cells with *Ct* serovar L2 displayed a separate anti-cLPS-mAb positive population presumably representing cells with inclusions (Figure 4 and Figure S4). The percentages of positive cells showed a broad range (from 25% up to 70%) depending on the MOI and the time of infection. For instance, after 40 h with MOI 10, 70.6% of the Jurkat- and 55.2% of the THP-1 reporter cells were *Ct* positive. Within these cells, suspected inclusion formation was detected in 55.3% of the Jurkat and 29.0% of the THP-1 reporter cells. These results obtained by standard flow cytometry were confirmed by imaging flow cytometry with inclusions accounting for 30.3% and 66.9% in the Jurkat reporter cells after 20 h and 40 h incubation, respectively (image flow data evaluation was performed as shown below for serovar E).

**Figure 5:**
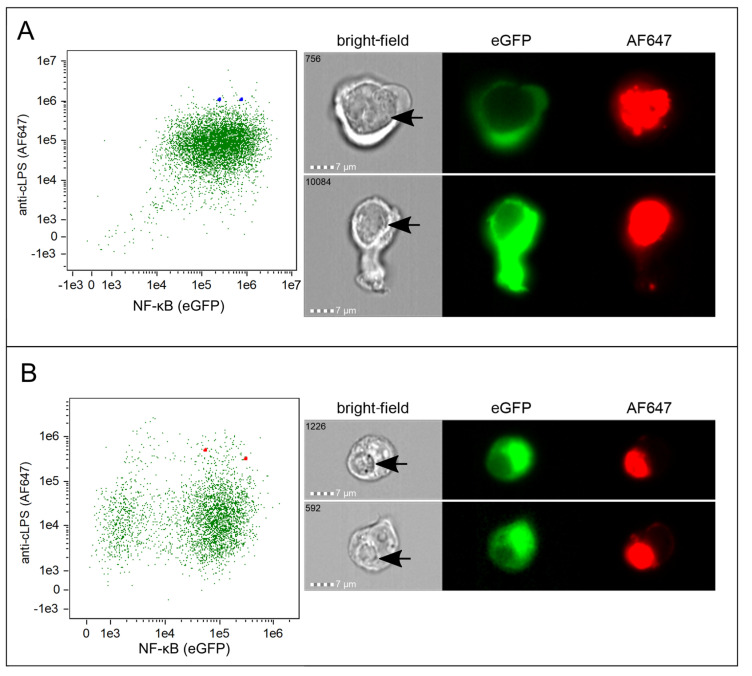
Imaging flow analysis of *Ct* incubated reporter cells. Representative dot plots including the corresponding imaging flow cytometry profiles of individual (**A**) THP-1 TLR4/CD14/MD2 reporter cells (blue squares) and (**B**) Jurkat TLR4/CD14/MD2 reporter cells (red squares) 40 h after inoculation with viable *Ct* serovar E (MOI 10). NF-κB signaling was measured by eGFP expression (x-axis) and uptake by intracellular staining with AF647-labelled anti-*c*LPS mAb (y-axis). The two selected cells indicated in the dot plots by the squares are shown in the bright-field, AF647 and eGFP channel. Inclusions are marked with black arrows in the bright-field. The sequence number of each individual cell recorded is indicated in the bright-field image.

In contrast to *Ct* serovar L2, standard flow cytometry of reporter cells infected with *Ct* serovar E did not indicate formation of inclusions (Figure 4 and Figure S4). However, imaging flow cytometry, which enabled the visualization of the corresponding image for each selected single cell, disclosed that serovar E also replicates within the myeloid (Figure 5A) as well as the T-cell reporter cells (Figure 5B). For comparison and as control, reporter cells incubated with UV-inactivated *Ct* or *Ct*-LPS showed no formation of inclusions (Figure S5).

The percentages of cells with both surface-bound as well as internalized cLPS varied depending on the CD14 co-receptor expression and the cell line. The THP TLR4/CD14/MD2 reporter cells displayed 91.4% cLPS positive cells, the Jurkat TLR4/CD14/MD2 reporters 70% and the Jurkat TLR4/MD2 cells the least (45.5%). Differences in internalization also depended strongly on the cell type and CD14 with 87.3% internalization of *Ct* in the THP TLR4/CD14/MD2 reporters and only 29.0% and 19.3% in the Jurkat TLR4/CD14/MD2 and Jurkat TLR4/MD2 reporters, respectively.

Imaging flow cytometry allowed us also to discriminate between growing *Ct* inclusions and non-growing cLPS positive entities (spots). The feature with the highest discrimination power between inclusions and spots was “median pixel” intensity of the cLPS signal with a Rd of at least 1.5 in all cell lines (Figure S6). By adding a size measurement of the cLPS signal as the second-best feature category (perimeter of a 50% threshold mask, Rd = 2.3, ranking highest in Jurkat TLR4/CD14/MD2), we succeeded in optimally separating the two populations. Inclusions were defined by a minimum perimeter of 20 µm and a median pixel intensity of more than 200 a.u. The distribution of inclusions was highest in the Jurkat TLR4/MD2 reporters (11.1%) in comparison to both THP-1 (9.5%) and Jurkat (8.5%) reporters expressing TLR4/CD14/MD2 (Figure S6C).

### 3.5. The Role of the TLR4/CD14/MD2 Complex in Ct Uptake 

**Figure 6:**
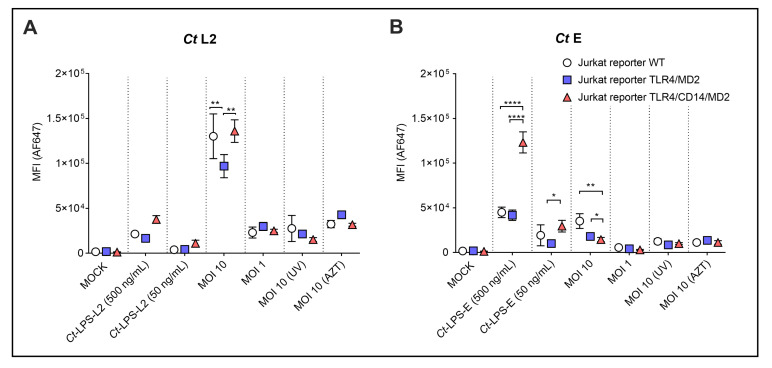
Analysis of uptake of *Ct* preparations by the different Jurkat reporter cells. WT, TLR4/MD2 or TLR4/CD14/MD2 Jurkat reporter cells were incubated for 20 h without or with monomeric *Ct*-LPS derived from the different *Ct serovars* (*Ct*-LPS-L2 or *Ct*-LPS-E), and serovar L2 (**A**) or serovar E (**B**) in viable- (MOI 10, MOI 1), UV-treated- [MOI 10 (UV)] or AZT-treated form [MOI 10 (AZT)]. The cells were stained intracellularly with the AF647-labelled anti-*c*LPS mAb 512F. The data represent means ± SEM of MFI of three independent experiments. Statistical differences between the reporter cell lines (WT, TLR4/MD2 and TLR4/CD14/MD2) and the different conditions were assessed by 2way ANOVA followed by Tukey’s multiple comparisons test. * *p* < 0.05, ** *p* < 0.01, **** *p* < 0.0001. Only statistically significant differences between conditions are indicated. Error bars represent mean ± SEM.

Finally, we analyzed the role of the TLR4/CD14/MD2 complex in the uptake of *Ct*. We did not see a significant difference between the MFI (anti-cLPS mAb) of the TLR4/CD14/MD2- and the WT THP-1 reporter cells upon incubation with *Ct* L2 and E (data not shown). However, the Jurkat reporters showed dependency on the CD14 co-receptor especially upon incubation with *Ct*-LPS-E (Figure 6).

*Ct*-LPS-L2 showed a similar profile, however, the uptake was much weaker (MFI 3.76 × 10^4^) compared to *Ct*-LPS-E (MFI 1.23 × 10^5^). Uptake of viable *Ct* serovar L2 (MOI 10) was high (MFI 1.36 × 10^5^) and to a certain extent independent of TLR4/MD2 and TLR4/CD14/MD2 expression (Figure 6A). However, the Jurkat reporters expressing TLR4/MD2 alone took up significantly less *Ct* serovar L2 than those cells with CD14 and the WT cells. This difference was not seen at a lower infectious dose (MOI 1). For viable *Ct* serovar E (MOI 10), the expression of TLR4/MD2 or the TLR4/CD14/MD2 complex resulted in a statistically significant reduction in the already weak MFI (3.53 × 10^4^ vs. 1.79 × 10^4^ or 1.45 × 10^4^, respectively). Compared to MOI 10, the *Ct* preparations MOI 1, UV-inactivated (MOI 10 UV) or AZT-treated (MOI 10 AZT) only provided a weak signal (MFI did not exceed 1.12 × 10^4^) without statistically significant differences between the various reporters.

## 4. Discussion

The importance of different PRRs in the recognition of *Ct* has been discussed previously [12,35,36,37,38]. While several reports indicate a prominent role of TLR2 in the recognition of *Ct,* the importance of TLR4 is addressed controversially [12,14,20]. Because of this shortage of information, we established a reporter cell system to dissect the role of the different cell surface bacteria-recognizing TLRs, i.e., TLR1/2, TLR2/6, TLR4, TLR4/CD14 and TLR5, regarding cellular uptake and induction of the NF-κB pathway through *Ct* and its main cell membrane component LPS. For this endeavor, we generated myeloid- and T-cell reporter cells expressing the different types of TLRs together with eGFP under the control of NF-κB. Further, although several studies already investigated the survival of *Chlamydia* spp. in myeloid cells [39,40,41,42], the role of lymphocytic cells as survival niche for replication and dissemination of *Ct* was not examined extensively [33]. Therefore, in addition to the role of TLRs, another aim of our study was to address the importance and capacity of lymphocytic cells for recognition, uptake and survival of *Ct* in comparison to myeloid cells. Finally, we investigated whether the latter parameters are differently embraced by serovars causing diverse clinical manifestations, namely serovar E causing urogenital infections and serovar L2 causing LGV.

Interestingly, when we incubated the myeloid THP-1 TLR4/CD14/MD2 reporters with monomeric *Ct*-LPS, only *Ct*-LPS from serovar L2, but not from E induced NF-κB. This is in contrast to previous findings, where no differences between serovars in the *Ct*-LPS mediated activation were detected [16]. One could argue that our experiment was not properly conducted, for instance that the *Ct*-LPS-E preparation we used was simply damaged. However, this was not the case, because *Ct*-LPS-E induced an even stronger NF-κB activation in the Jurkat T-cell TLR4/CD14/MD2 reporter cells than *Ct*-LPS-L2 from serovar L2. An explanation for these seemingly controversial findings was shown previously in the study authored by Yang et al. [39]. There, the authors described a CD14-mediated endocytosis, yet inefficient dimerization of TLR4/MD2 by *Ct*-LPS-E. This was supported by our study, in which *Ct*-LPS-E uptake in the Jurkat TLR4/CD14/MD2 reporters was more pronounced than the uptake of *Ct*-LPS-L2. However, the study by Yang et al. only addressed the inflammatory properties of *Ct*-LPS-E and not of *Ct*-LPS-L2. Thus, our findings demonstrate a variation in uptake, as well as signaling between both serovars. A tempting interpretation of these results would be the attempt of an escape mechanism mediated by either an unknown host factor - or a factor expressed by serovar E to ignore or to inhibit the crucial defense pathway NF-κB in the monocytic cells. Furthermore, another possibility in the differential recognition of *Ct*-LPS-E and -L2 is the subsequent signaling cascade through MyD88- or TRIF-dependent pathways. Signaling by MyD88 is mediated directly by surface dimerized TLR4/MD-2 [39] and may be important in the recognition of *Ct*-LPS of both serovars by Jurkat reporters. In contrast, the prerequisite for activation of the TRIF-dependent pathway is the CD14-dependent endocytosis [40], which might be important in the recognition of *Ct*-LPS-E in myeloid cells, especially when an insufficient TLR4/MD2 dimerization on the cell surface was induced. Due to CD14 internalization and *Ct*-uptake, this may also result in an escape-mechanism, resulting in an insufficient activation of the MyD88-pathway and signaling by *Ct*-LPS-E. However, immune cells obviously developed a counterstrategy, because both the myeloid- and the T-cells control serovar E better than L2 in terms of uptake and formation of inclusions. Evidently involved in this defense mechanism are TLR4/MD2 and CD14, because Jurkat cells expressing TLR4/CD14/MD2 become significantly less infected than WT Jurkat cells. These finding are corroborated by imaging flow cytometry that allowed us to discriminate between simple uptake of *Ct* and growth of inclusions (Figure S6C). Irrespective of this uptake dependency of serovar E on TLR4/MD2 and CD14, this result also shows that uptake of *Ct-*LPS and NF-κB activation regardless of the serovar is decoupled from uptake and growth of intact *Ct*, which obviously highjack other cellular receptors than TLR1/2, TLR2/6, TLR4/MD2 and TLR4/CD14/MD2 for this process. WT Jurkat cells, which do not constitutively express these TLRs, were always higher (in terms of TLR4/CD14) or similarly (in terms of TLR1/2, TLR2/6) positive with both serovars than the reporter cells ectopically expressing these TLRs. The essential role of CD14 in the recognition of *Ct* is indicated because NF-κB activation was detectable only in Jurkat reporter cells expressing TLR4/CD14/MD2, but not in those expressing TLR4/MD2 without CD14. This finding is in accordance with a study describing dependency on CD14 by TLR4 recognition [16]. Though, in contrast to that study our findings demonstrate that monomeric *Ct*-LPS is almost as effective in inducing NF-κB signaling as is *E. coli* LPS. Nonetheless, for NF-κB activation by the latter, CD14 is not essential and TLR4/MD2 alone is sufficient. However, this contrasts with the study by Yang et al., in which *Ct*-LPS-E was demonstrated to show a lack of NF-κB and IRF3 phosphorylation [39]. Of note are the different responses of the Jurkat- and the THP-1 reporters towards *Ct*-LPS-E and *Ct*-LPS-L2. Further, our results suggest a different uptake-mechanism between intact *Ct* and monomeric *Ct*-LPS. Therefore, alternative cell-type dependent factors than CD14 may be involved in the uptake of *Ct-*LPS-E and NF-κB signaling.

By gathering the specific flow cytometry profiles after incubation of the myeloid- and T-cell reporters with *Ct*, we identified variable patterns depending on the serovar as well as on the cell line. The signal intensity in each channel provided information about the uptake of intact *Ct* or *Ct-*LPS as well as the recognition by the TLR4/CD14/MD2 complex through measuring NF-κB. As described above, the most prominent difference in signaling by measuring NF-κB was detected between the myeloid and lymphocytic cell lines when comparing *Ct*-LPS. Here, the Jurkat cell line was shown to be more efficient in recognizing *Ct*-LPS. Furthermore, the setup of this infectivity assay also provided the possibility to easily monitor NF-κB production intensity and its dependency on *Ct* uptake. A strong uptake signal was shown after incubation of both cell lines with viable *Ct* serovar L2. Because of this signal and the development of a separate population, it is tempting to speculate that the pathogen survives and replicates within these cells. In contrast to serovar L2, the flow cytometry profiles of serovar E did not show differences in terms of signal intensity between viable and inactivated preparations and, therefore, no evidence of effective replication. But, a corresponding imaging flow analysis as proposed recently for the analysis of intracellular pathogens [31] revealed that also serovar E is able to replicate and form *Ct* inclusions in both cell types. By this approach, we were also able to calculate the number of infected cells by comparing inclusions versus non replicating “spots” and computed that *Ct* serovar E in comparison to serovar L2 forms approximately 8-fold less inclusions in both the Jurkat- and the THP-1 reporter cells expressing the TLR4/CD14/MD2 complex. This finding is in agreement with the recently described cell-type and *Ct* serovar dependence of cytokine and chemokine production and the strong growth of serovar L2 but not of serovars A and D in THP-1 cells [34].

The specific structure of *Ct-*LPS with less potent capacity compared to *E. coli* LPS in TLR4 binding or activation was already discussed [16,41,42], and the questionable role of TLR4 in *Ct* recognition was investigated before not only in animal models [12] but also in clinical studies [43,44,45,46]. However, in contrast to these previous studies and to *E. coli* LPS, for which the TLR4/MD2-complex was sufficient, by using the Jurkat reporter cells we demonstrate the decisive function of CD14 in the TLR4/CD14/MD2-complex for *Ct-*LPS recognition, cellular uptake and NF-κB signaling. The NF-κB signaling was not mediated by other TLRs, since neither WT THP-1 reporter cells constitutively equipped with TLR1/2, TLR2/6 and TLR5 nor Jurkat reporter cells ectopically expressing one of these TLRs alone revealed NF-κB-mediated eGFP expression. Thus, the TLR4/CD14/MD2 complex appears to play a significant role in the recognition of this pathogen via *Ct*-LPS for NF-κB signaling. With the WT Jurkat cells, however, we also clearly show that for binding and uptake of *Ct,* this complex plays only an additive role. Several host cell receptors are known to bind *Ct* including heparan sulfate proteoglycans, β1 integrin, the epidermal growth factor receptor, and the mannose 6-phosphate receptor (CD222) [47]. With the exception of CD222 - which we excluded, because we knocked it out in the Jurkat cells via CRIPSR-Cas9 and did not find a reduced or altered infection rate (data not shown)–we currently do not know whether one of these known or an unknown receptor is used by *Ct* to infect our reporter cells. Although, it was previously described that CD14 mediates endocytosis of TLR4, as well as the internalization of extracellular macromolecules in a LPS-dependent manner [48], we did not observe this in the case of *Ct*. Such CD14-mediated endocytosis possibly could have initiated an indirect uptake-mechanism of *Ct*. However, we only detected in case of monomeric *Ct*-LPS-E a dependency upon CD14 expression that enabled *Ct*-LPS-E uptake in our cell lines. Despite the missing effect on the internalization of whole *Ct* preparations and, interestingly, also monomeric *Ct*-LPS-L2, further investigations are needed to confirm these results and find explanations for the potential serovar-dependent differences in CD14 mediated endocytosis of *Ct*-LPS.

## 5. Conclusions

To conclude, we pinned down the TLR4/CD14/MD2 complex as the only surface TLR recognizing *Ct* for activating the NF-κB pathway. We also found serovar-specific differences in NF-κB activation as well as infection. The ignorance of *Ct-*LPS-E but not of *Ct*-LPS-L2 by the TLR4/CD14/MD2 complex for NF-κB activation in the monocytic THP-1 cells can be interpreted as part of an immune escape strategy. On the other hand, we clearly show that serovar L2 in contrast to serovar E has an 8-fold higher competence to infect and grow in both the monocytic and the lymphoid cells. Although the underlying mechanism of this difference is not yet clear, this feature of serovar L2 to potently infect immune cells may explain the dissemination of the LGV biovar into lymph nodes causing the clinical manifestations of LGV.

## Data Availability

The raw data supporting the conclusions of this article will be made available by the authors, without undue reservation.

## Supplementary Materials

The following supporting information can be downloaded at: https://www.mdpi.com/article/10.3390/microorganisms10122489/s1, Figure S1: Overview of the reporter cell lines used in this study; Figure S2: NF-κB induced expression of eGFP in Jurkat TLR4/CD14/MD2 reporter cells upon 40 h incubation; Figure S3: Flow cytometry histograms displaying NF-κB-eGFP expression (standard logarithmic scale) in Jurkat reporter cells ectopically expressing either TLR1/2/6 or TLR2/6 after stimulation with specific TLR ligands and Ct serovar E; Figure S4: Flow cytometric analysis of signaling as well as uptake of Ct by (A) THP-1 TLR4/CD14/MD2 and (B) Jurkat TLR4/CD14/MD2 reporter cells; Figure S5: Imaging flow internalization assays of reporter cells; Figure S6: Discrimination of Ct inclusions versus spots by imaging flow cytometry.
