# Supplementary material for: TLR4/CD14/MD2 Revealed as the Limited Toll-like Receptor Complex for Chlamydia trachomatis-Induced NF-κB Signaling
Source: Microorganisms. 2022 Dec 15;10(12):2489. doi: 10.3390/microorganisms10122489 (PMC9783372; doi:10.3390/microorganisms10122489)
Supplement: Supplementary file 1 [file microorganisms-10-02489-s001.zip › microorganisms-2048442-supplementary.pdf]

microorganisms

Article

TLR4/CD14/MD2 Revealed as the Limited Toll-like Receptor Complex for *Chlamydia trachomatis*-Induced NF-κB Signaling

Supplementary material

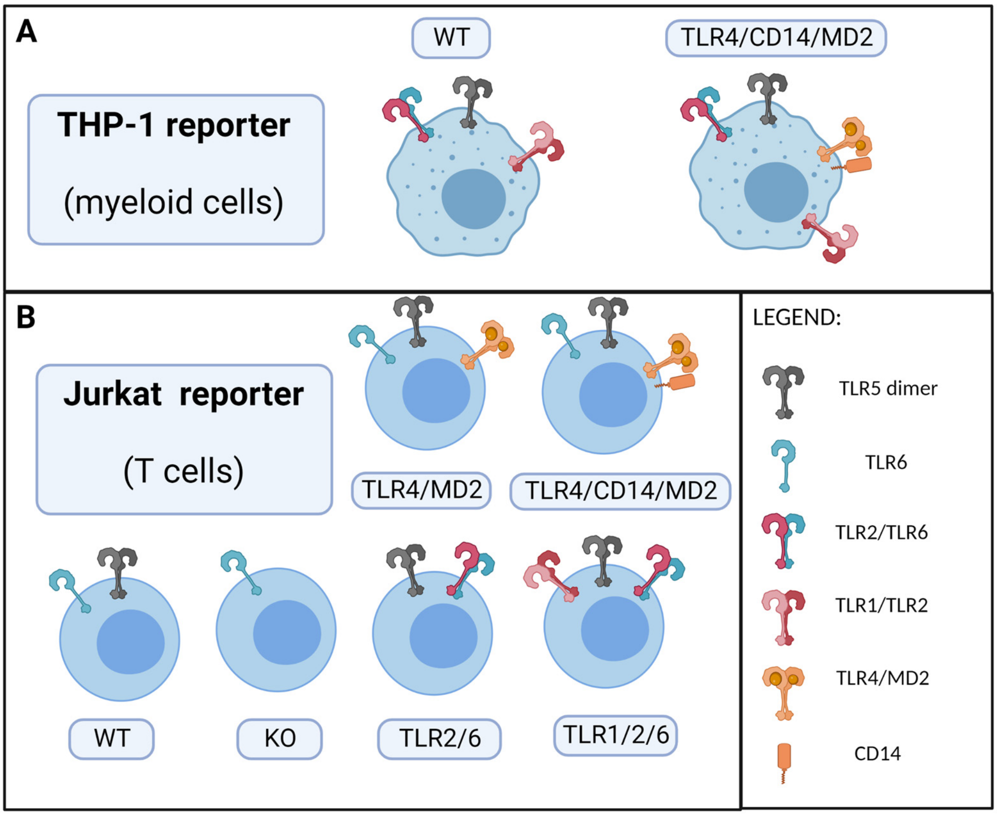

Figure S1. Overview of the reporter cell lines used in this study. (A) THP-1 reporter cells: WT (THP-1 cells naturally expressing TLR1/2, TLR5 and TLR2/6), TLR4/CD14/MD2 (THP-1 cells ectopically expressing TLR4/CD14/MD2). (B) Jurkat reporter cells: WT (Jurkat clone E6-1 cells naturally expressing a TLR5 dimer and a non-functional TLR6 monomer), KO (Jurkat clone E6-1 TLR5 knock-out cells generated by using the CRISPR/Cas9 system), TLR2/6 (Jurkat E6-1 cells ectopically expressing TLR2 resulting together with endogenous TLR6 in expression of functional TLR2/6), TLR1/2/6 (Jurkat E6-1 cells ectopically expressing TLR1 and TLR2 resulting together with endogenous TLR6 in expression of functional TLR1/2 as well as functional TLR2/6), TLR4/MD2 (Jurkat E6-1 cells ectopically expressing TLR4 and MD2), TLR4/CD14/MD2 (Jurkat E6-1 cells ectopically expressing TLR4, CD14 and MD2). Created with BioRender.com.

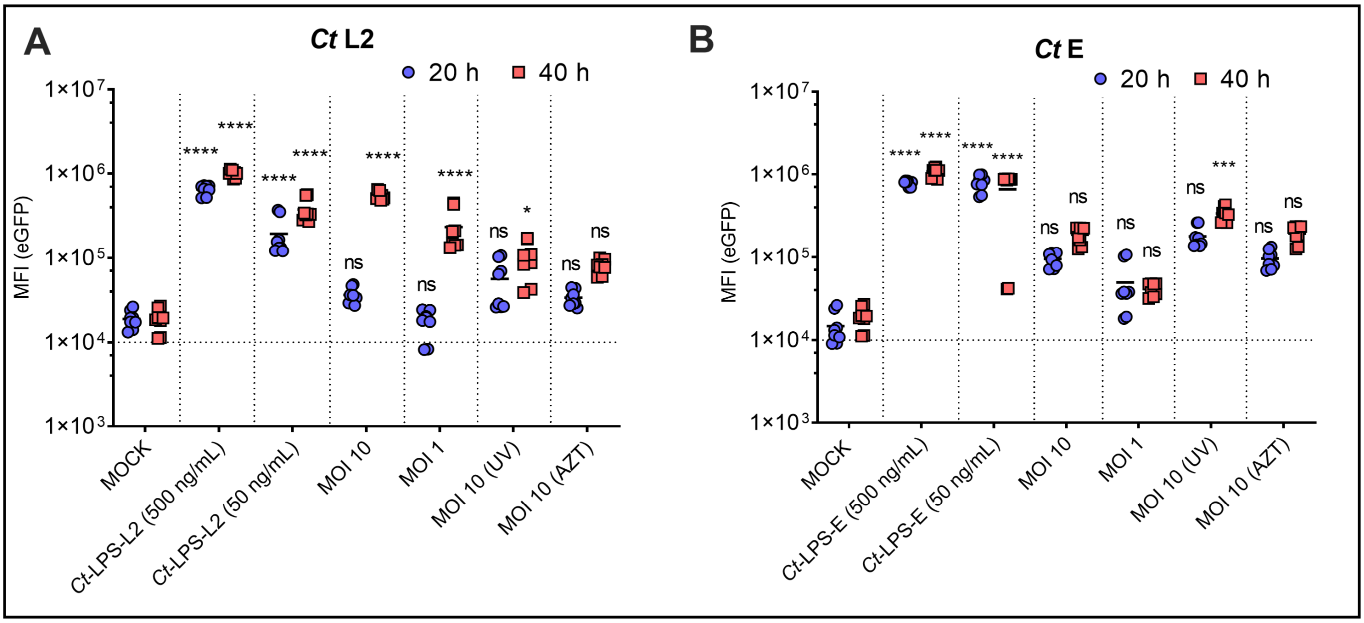

Figure S2. NF-κB induced expression of eGFP in Jurkat TLR4/CD14/MD2 reporter cells upon 40 hours incubation. *Ct* preparations derived from serovar L2 (A) or serovar E (B) were incubated for 20 hours or 40 hours with TLR4/CD14/MD2 Jurkat reporter cells. *Ct*-LPS-L2 – *Ct* serovar L2 derived LPS, *Ct*-LPS-E – *Ct* serovar E derived LPS, MOI 10 (UV) – UV-treated *Ct* with MOI 10, MOI 10 (AZT) – AZT-treated *Ct* with MOI 10. Data represent eGFP-MFI means ± SEM of three independent experiments. Statistical differences between mock and the different conditions were assessed by 2way ANOVA followed by Dunnett's multiple comparisons test. *P < 0.05, **P < 0.01, ****P < 0.0001; ns, not significant. Individual measurements are shown (n = 8), each experiment was performed in duplicates.

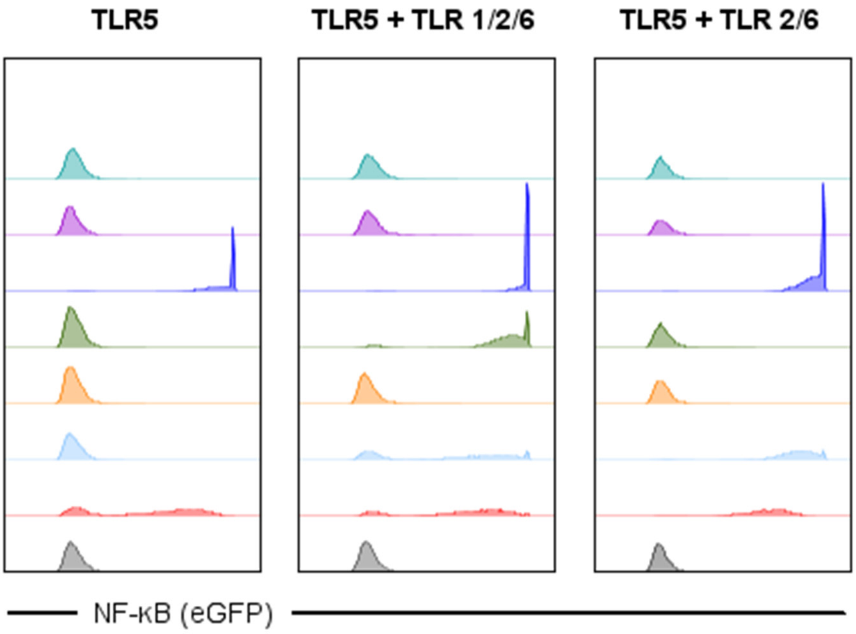

Figure S3. Flow cytometry histograms displaying NF-κB-eGFP expression (standard logarithmic scale) in Jurkat reporter cells ectopically expressing either TLR1/2/6 or TLR2/6 after stimulation with specific TLR ligands and *Ct* serovar E. Measurements were performed after 24 hours incubation with medium only (grey, mock control) TLR5 ligand flagellin 100 ng/mL (red, Jurkat cells express constitutively TLR5), TLR2/6 ligand FSL-1 100 ng/mL (light blue), TLR4 ligand LPS 50 ng/mL (orange), TLR2/1 ligand Pam3CSK4 100 ng/mL (green), PMA/ionomycin 100 nM (dark blue, positive control), *Ct* serovar E MOI 10 (violet) and *Ct* serovar E MOI 1 (turquoise).

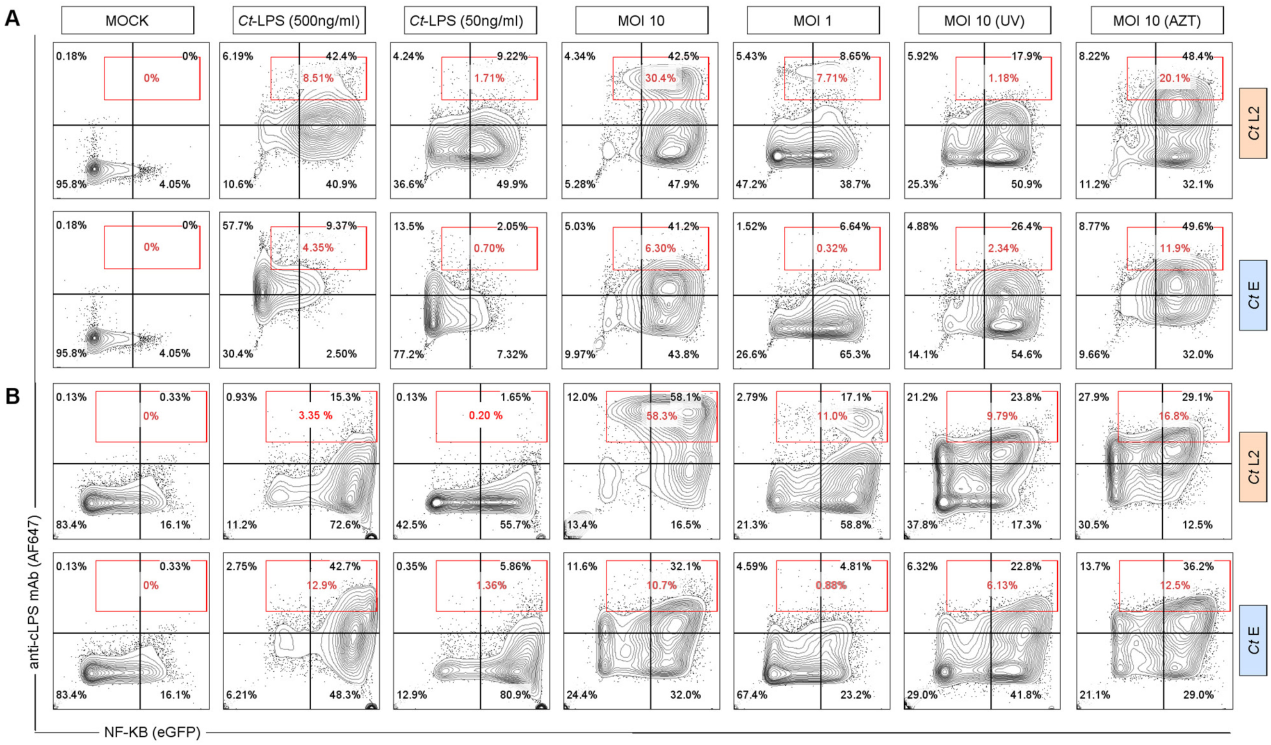

Figure S4. Flow cytometric analysis of signaling as well as uptake of *Ct* by (A) THP-1 TLR4/CD14/MD2 and (B) Jurkat TLR4/CD14/MD2 reporter cells. Representative contour plots of NF-κB signaling measured by eGFP intensity (x-axis) and uptake measured by intracellular staining with anti-cLPS mAb (y-axis) of the reporter cells. The cells were incubated for 40 hours with *Ct* preparations of serovar E (*Ct* E) or serovar L2 (*Ct* L2). Mock controls are shown in the first row and compared to cells treated with the various *Ct* derived preparations. The preparations included viable bacteria (MOI 10, MOI 1) as well as UV- [(MOI 10 (UV)] and AZT-treated [(MOI 10 (AZT)] *Ct*. LPS of each serovar (*Ct*-LPS-L2 or *Ct*-LPS-E) was used at 2 different concentrations and the respective one is displayed in the row corresponding to its *Ct* serovar (*Ct*-LPS). The percentages of cells in each quadrant are shown and the fluorescence cut-off was set at 0.2-0.45% of the mock. An additional gate (red) indicates the location of cells with high probability of replicating *Ct*.

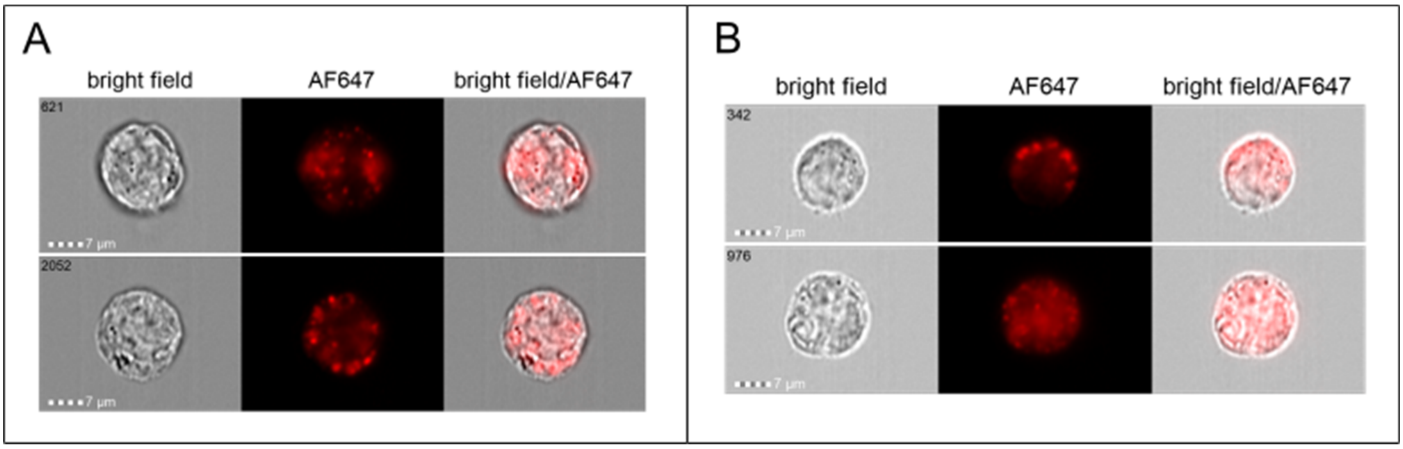

Figure S5. Imaging flow internalization assays of reporter cells. Representative images of THP1 TLR4/CD14/MD2 reporter cells 40 hours after inoculation with (A) UV inactivated preparations of *Ct* serovar E [(MOI 10 (UV)] or (B) *Ct*-LPS-E are shown. The sequence number of each individual cell recorded is indicated in the bright field image.

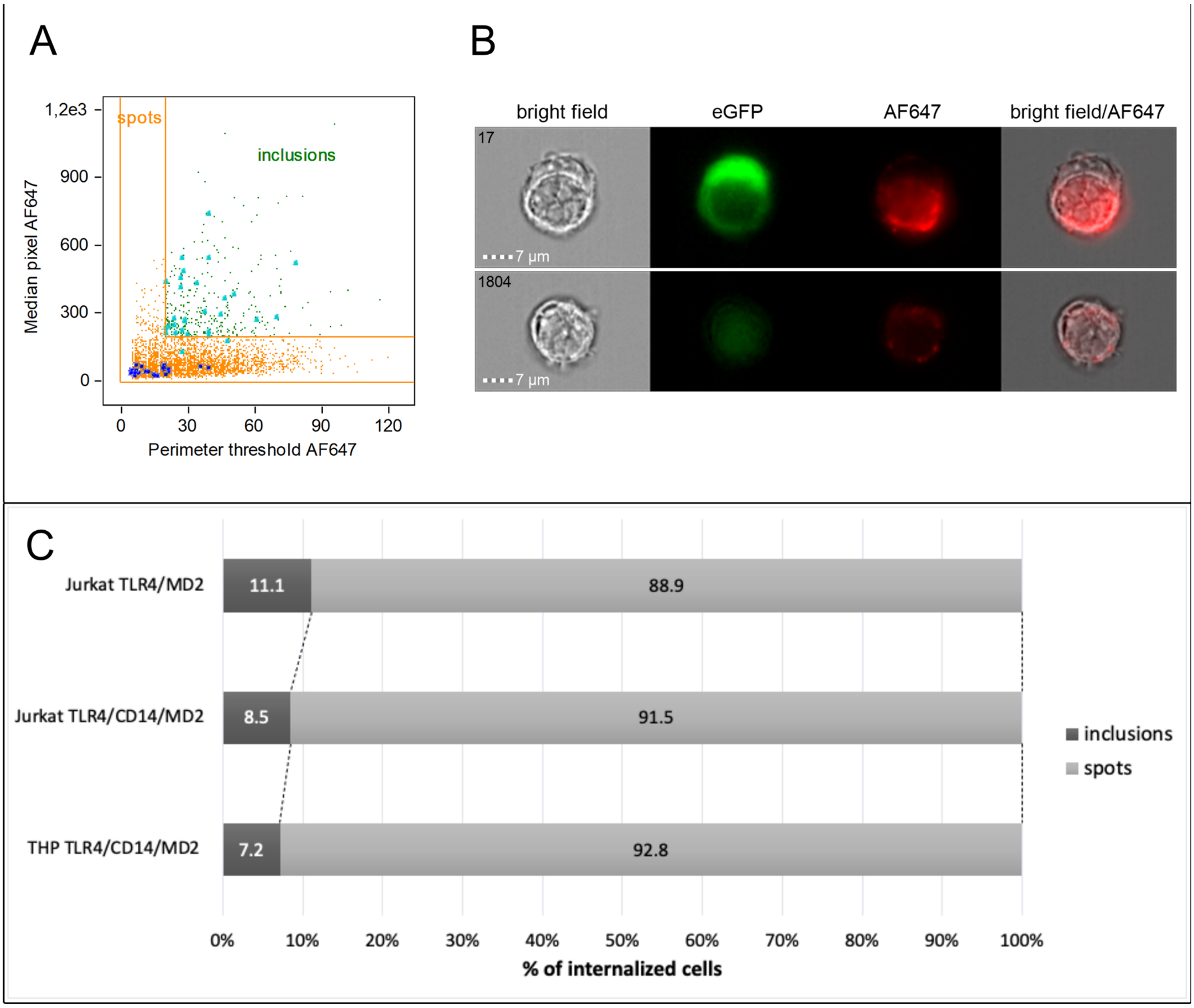

Figure S6. Discrimination of *Ct* inclusions versus spots by imaging flow cytometry. THP1 TLR4/CD14/MD2 reporter cells were incubated for 40 hours with *Ct* serovar E. Afterwards, the cells were permeabilized and stained using AF647-labelled anti-cLPS mAb 512F. (A) For discrimination of spots versus inclusions the IDEAS® guided analysis for feature finding was utilized. The best discrimination between the two morphologically different populations (inclusions versus spots) was achieved considering the median pixel intensity and the perimeter of the cLPS (AF647) signal. Inclusions were defined by a minimum perimeter of 20 µm and a median pixel intensity of more than 200 a.u.. The statistical separation of both populations was calculated with the Fisher's Discriminant Ratio. Individual cells selected by the feature finder as spots are shown in the dot plot as blue squares, cells with inclusions as turquoise squares. Only single focused cLPS positive cells were included in the analysis. **(B)** Representative images of cells with inclusions (upper row) and spots (lower row) chosen for the Feature Finder analysis are shown. The sequence number of each individual cell recorded is indicated in the bright field image. **(C)** Calculation of distribution of inclusions and spots within internalized *Ct* positive cells. Data represent percentages of at least 5000 analyzed cells.
